# Editorial: Advancing coaching scholarship

**DOI:** 10.3389/fpsyg.2025.1544495

**Published:** 2025-03-13

**Authors:** Sewon Kim, Rajashi Ghosh, Rob Poell, Terrence E. Maltbia

**Affiliations:** ^1^Department of Business Management and Leadership, The State University of New York (SUNY) | Empire State, New York, NY, United States; ^2^Department of Organization and Leadership, Columbia University, New York City, NY, United States; ^3^Department of Human Resource Studies, Tilburg University, Tilburg, Netherlands

**Keywords:** executive coaching, managerial coaching, meta-analysis, artificial intelligence (AI), digital coaching, coaching scale, humor

The popularity of coaching in the workplace has markedly increased over recent years. According to the International Coaching Federation's (ICF) 2023 Global Coaching Study, the global coaching market has an estimated value of ~$5.34 billion as of 2023 and is projected to grow to about $6.25 billion in 2024. The study also reported around 126,000 active coach practitioners worldwide in 2023, with projections indicating this number could reach 145,500 by 2024. This data shows significant growth, with the number of coaches nearly doubling since 2019, when there were roughly 71,000 coaches globally. IBISWorld's statistics show that as of 2024 the market size of the Business Coaching industry is $14.1 billion, an increase of −0.47% from 2023 (i.e., includes executive coaching and leadership development). The Chartered Institute of Personnel and Development's (CIPD) studies report that substantial investment is being made to build internal coaching and human capital capacity within work organizations and the number of managers utilizing coaching skills continues to rise sharply.

Despite this growth, academic research on coaching trails behind the practice of coaching. Much of the present knowledge is derived from best practices or opinion-based approaches rather than scientific investigation. We lack an understanding of the theories and foundational concepts that shed light on coaching practice. The existing gap between practice and research hinders our full understanding of the real impact and applications of coaching. Our Research Topic aims to bridge this gap by featuring six empirical articles that investigate various dimensions of workplace coaching. The current editorial showcases these scientific contributions and discusses implications for coaching practice and research.

## Contextualizing coaching research

As coaching becomes a key developmental tool for executives, managers, and employees, the call for a solid theoretical and empirical basis is on the rise. Scholars have underscored the need for a theoretical basis that enhances the practice of coaching and the importance of exploring coaching mechanisms across both conventional and alternative forms (Boyatzis et al., 2022). This Research Topic responds to that call. Herein, we present research studies that explore executive coaching effectiveness, digital coaching, artificial intelligence (AI) in coaching, managerial behaviors and beliefs, and the impact of humor in the professional coaching process.

The Research Topic presents theoretical and empirical articles of both relevance and rigor in the scientific investigation of coaching. Across the six articles, the Research Topic introduces theoretical frameworks and underpinnings that can ground and exemplify coaching practice. Spanning various topical areas, these articles offer an empirical base of original research on different types and approaches to coaching. Through these contributions, this Research Topic consolidates and synthesizes extant academic research on coaching and provides implications for advanced coaching practice as well as future research agenda.

## Contributing articles

The six articles in this Research Topic tackle different aspects of coaching, adding valuable insights into the field:

1. **Meta-analysis of randomized controlled trial studies on executive coaching effectiveness** (Nicolau et al.): this article brings clarity to the psychological dimensions that are most impacted by executive coaching, advancing our understanding of how executive coaching can be used by managers to increase performance in organizations. The authors conducted a meta-analysis of 20 studies that employed rigorous experimental designs and found that the impact of coaching on behavioral outcomes was higher compared to other person characteristics outcomes, indicating that cognitive behavioral activities are most likely to be impacted by executive coaching.
2. **Defining digital coaching** (Diller and Passmore): digital coaching is gaining prominence in organizations. Employing a qualitative, inductive approach, the authors surveyed 260 coaches practicing digital coaching and explored what digital coaching is all about. The study participants defined digital coaching as ‘a digital-technology-enabled, synchronous conversation between a human coach and a coachee', and differentiated it from AI coaching and other digital-technology-enabled development formats. The study offers a conceptual basis for future investigations into the characteristics, processes, and quality of digital coaching.
3. **AI vs. human coaches** (Barger): using a mixed-methods randomized controlled trial, this study examines client perceptions of AI coaching in comparison to human coaching. The authors employed alternative treatment groups of AI and human coaches, as well as a control group. Results show that clients can form equally strong work relationships with both types of coaches. Qualitative themes challenge traditional views on AI's limitations and highlight its potential applications in future coaching practices. The study presents new possibilities for how AI could support and extend coaching engagements.
4. **Managerial coaches' enacted behaviors and the beliefs that guide them** (Adele and Ellinger): using the critical incident technique and combining data from both coaches and coachees, this study presents coaching behaviors that managers enact along with a comprehensive understanding of their guiding beliefs. Rich descriptions illustrate the findings and implications for theory, research, and practice are also discussed.
5. **Examining the predictive validity of a managerial coaching scale** (Stone et al.): this study assesses the predictive validity of the widely used Employee Perceptions of Supervisor/Line Manager Coaching Behavior Measure managerial coaching scale (CBI) using a longitudinal design. It highlights the complex relationships between managerial coaching and various workplace outcomes.
6. **Humor in professional coaching** (Vendl et al.): this research focuses on the use of humor in coaching dialogues. The authors conducted a systematic literature review on humor and drew insights from counseling, psychotherapy, mentoring, and related fields. They argue that humor can be an effective tool for strengthening coaching processes and interactions. The study proposes that coach training and education incorporate humor as a valuable strategy for enhancing the coaching alliance.

## Implications

The articles presented in our Research Topic collectively contribute to the advancement of coaching. They illuminate the multifaceted nature and richness of coaching and provide empirical evidence for coaching across both traditional and alternative practice formats. The studies demonstrate that workplace coaching practice is shaped by various elements, including the coach's beliefs and methods, the attitudes of the clients they coach, the channel through which coaching is conveyed, and the use of humor in coaching interactions. They also suggest that simulated AI coaches have the potential to be valuable complements to human coaches. Together, these findings signal new possibilities for coaching in the workplace.

In conclusion, this Research Topic of *Frontiers in Psychology* highlights the evolving landscape of coaching scholarship, emphasizing its transformative role in both individual and organizational development. The featured research advances our understanding of coaching practices, methodologies, and outcomes, with implications for practitioners and academics alike. Future research could explore questions such as: How can coaching more effectively integrate with hybrid intelligence? What mechanisms most impact sustained behavioral change in those being coached? And, how can coaching adapt to diverse cultural and digital contexts? These questions open pathways for continued investigation in advancing coaching as a dynamic field.
